# Risk Factors of Impaired Perfusion in Patients With Symptomatic Internal Carotid Artery Steno-Occlusive Disease

**DOI:** 10.3389/fneur.2022.801413

**Published:** 2022-04-14

**Authors:** Xinxin Qiao, Jinfeng Duan, Nan Zhang, Yang Duan, Xinrui Wang, Yusong Pei, Zhihua Xu, Benqiang Yang, Miao Qi, Jinze Li

**Affiliations:** ^1^Department of Radiology, The General Hospital of Northern Theatre Command, Shenyang, China; ^2^Jinzhou Medical University General Hospital of Northern Theatre Command Postgraduate Training Base, Shenyang, China; ^3^Department of General Surgery, The General Hospital of Northern Theatre Command, Shenyang, China; ^4^Department of General Surgery, Shengjing Hospital of China Medical University, Shenyang, China; ^5^Department of Radiology, TongDe Hospital of Zhejiang Province, Hangzhou, China

**Keywords:** internal carotid artery, CT perfusion, impaired perfusion, internal watershed, circle of Willis

## Abstract

**Objective:**

To quantitatively evaluate the impaired perfusion status of patients with symptomatic internal carotid artery (ICA) steno-occlusive disease and to explore the risk factors of impaired perfusion with computed tomography perfusion (CTP).

**Methods:**

The clinical and imaging data of 187 patients with ICA steno-occlusive disease were retrospectively analyzed. The ICA stenosis rate was divided into Grades I–IV (70–79%; 80–89%; 90–99%; 100%), and the circle of Willis was classified as four types (types I–IV). According to the literature, the value of cerebral blood flow/cerebral blood volume (CBF/CBV) of 7.55/min was used as cut-off to predict symptomatic patients. All patients were categorized into two groups: those with impaired perfusion [*n* = 99 (52.9%)] and those without impaired perfusion [*n* = 88 (47.1%)]. Symmetrical bilateral internal watershed areas were selected as the regions of interest (ROIs). Statistical analysis was made on the status of impaired perfusion and the risk factors of impaired perfusion.

**Results:**

Univariate analysis revealed that systolic blood pressure (SBP), diastolic blood pressure (DBP), mean arterial pressure (MAP), types of the circle of Willis, and clinical features at admission differed between the two groups (patients with or without impaired perfusion) (*p* < 0.05). Multiple logistic stepwise regression analysis showed that MAP [odds ratio (OR) = 0.946, 95% confidential interval (CI) = 0.917–0.974, *p* < 0.001] and type IV (type I vs. IV: OR = 4.987, 95% CI = 1.955–12.723, *p* = 0.001) at admission were independently associated with impaired perfusion in the internal watershed areas.

**Conclusion:**

MAP and the type of circle of Willis at admission are independent risk factors associated with the impaired perfusion in patients with ICA steno-occlusive disease.

## Introduction

Internal carotid artery (ICA) steno-occlusive disease is a leading cause of cerebral ischemia or infarction, especially in the internal watershed areas (1–3). Watershed brain tissues are more sensitive to impaired perfusion than other brain tissues, with the hypoperfusion state being the basis of its pathogenesis. Clinicians quantify the degree of perfusion impairment to identify early brain tissue injury status, transient cerebral ischemia (TIA), or acute brain infraction.

Several studies have identified that hemodynamic impairment of patients with severe stenosis or occlusion of ICA can predict the occurrence of stroke (4–6). Several studies have demonstrated that a decreased cerebral blood flow/cerebral blood volume (CBF/CBV) (<7.6/min) is a validated risk factor for symptomatic stroke (4, 7, 8). Computed tomography perfusion (CTP) is considered to have a similar diagnostic value to positron emission tomography (PET), which is the gold standard for evaluating cerebral hemodynamic impairment (9). CTP is sensitive in finding the abnormal perfusion area in the early stages of cerebral infarction, and can provide relevant hemodynamic functional information through various parameters and their ratios, which is helpful for clinicians to formulate an appropriate treatment plan for patients with ICA steno-occlusive disease. Patients with ICA steno-occlusive disease exhibiting hemodynamic impairment and insufficient collateral circulation could benefit from vascular recanalization surgery (10, 11).

Previous studies mainly focused on predicting the risk factors of stroke, but reports on risk factors affecting brain perfusion are relatively scarce. Moreover, the relationship between the circle of Willis and impaired perfusion had not been adequately investigated. Thus, we aimed to identify the independent risk factors of the impaired perfusion in the internal watershed areas induced by the occurrence of unilateral ICA steno-occlusive disease. This may help to determine the ideal candidates for further intravascular therapy.

## Materials and Methods

### Patients

**Figure 1:**
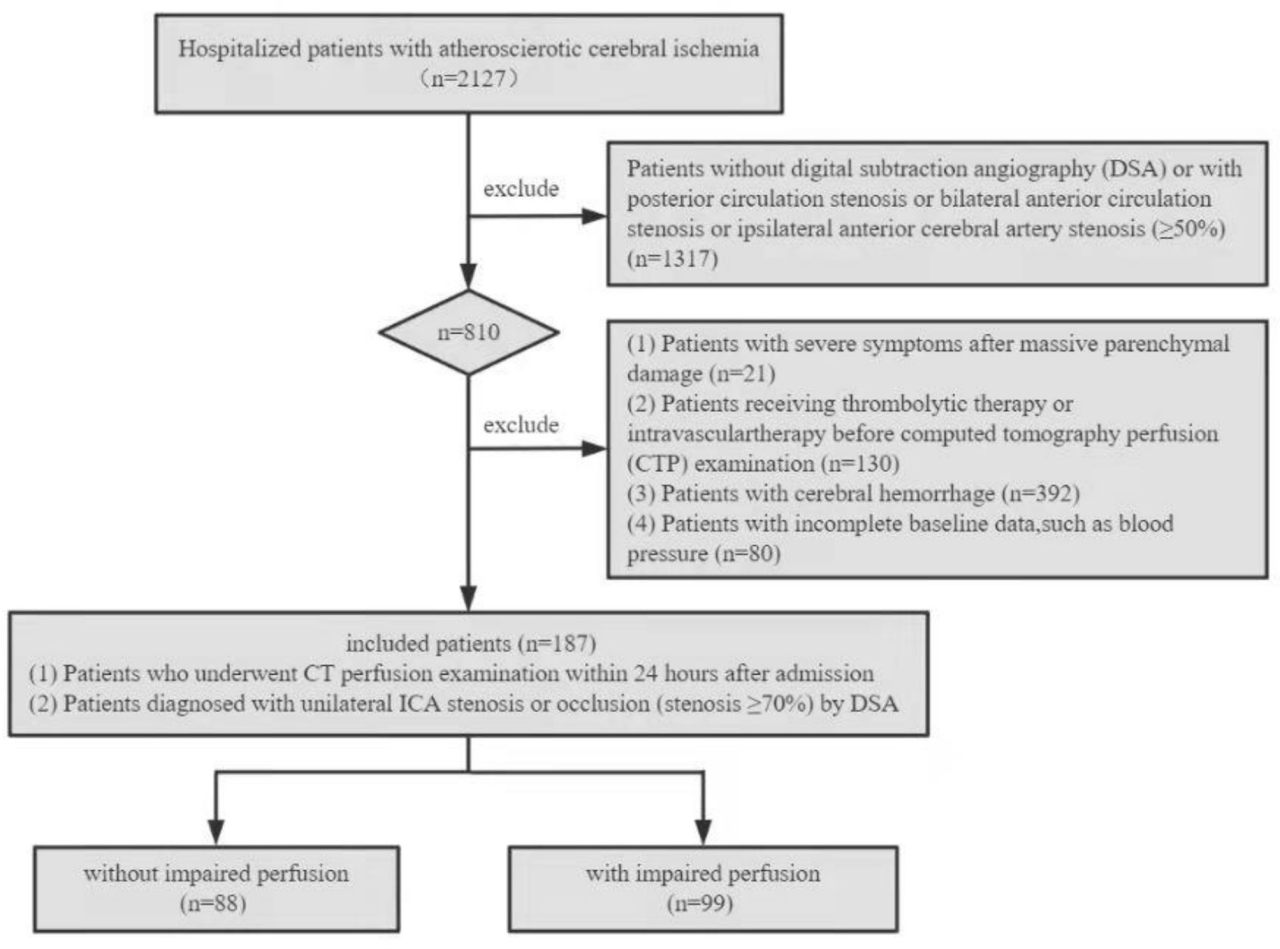
Flowchart of study population enrollment.

This retrospective study was approved by the Institutional Review Board of the General Hospital of the Northern Theater Command, and the study patients or their family members provided written informed consent. The clinical and imaging data of patients with unilateral ICA steno-occlusive disease from 1 January 2019 to 30 December 2021 were collected and reviewed. The inclusion criteria were specified as follows: (1) patients who underwent CTP within 24 h after admission and (2) patients with unilateral ICA stenosis or occlusion (stenosis ≥70%) diagnosed by digital subtraction anwwgiography (DSA). The following exclusion criteria were specified: (1) patients without DSA or with posterior circulation stenosis or bilateral anterior circulation stenosis or ipsilateral anterior cerebral artery (ACA) stenosis (≥50%); (2) patients with severe symptoms after massive parenchymal damage; (3) patients with previous intravenous or intra-arterial thrombolytic therapy, intracranial or extracranial arterial angioplasty, or stent implantation before CTP; (4) cerebral hemorrhage; and (5) patients with incomplete baseline data, such as blood pressure (BP). Figure 1 shows the flow chart of patient enrollment in this study.

### Clinical Information

The following data were collected from each study patient: age, sex, the presence of hypertension, hyperlipidemia, or hyperhomocysteinemia (HHcy), history of diabetes, coronary heart disease (CHD), atrial fibrillation (AF), stroke, current smoking status, alcohol use, left ventricular ejection fraction (LVEF), and admission BP [within 24 h before CTP, including systolic blood pressure (SBP), diastolic blood pressure (DBP), and mean arterial pressure (MAP)]. According to the clinical features, patients were divided into two groups: those in the symptomatic group with TIA or contralateral limb dysfunction within 6 months, and those in the asymptomatic group with no definite symptoms of nervous system ischemia or only mild discomfort (12).

### Imaging Protocols

Computed tomography perfusion examination was performed using a 64-slice spiral CT system (GE Discovery CT 750 HD, GE, USA), and the auditory-canthus-line was used as the baseline for scanning. A high-pressure injection indwelling needle was placed in the right cubital vein, and a high-pressure syringe was connected for plain CT and CTP. An injection of 40 ml iohexol (350 mg/ml, Xenetix; Guerbet, Aulnay-sous-Bois, France) and 20 ml normal saline was delivered through a high-pressure syringe at 5 ml/s through the cubital vein for CTP imaging. The scanning parameters used included: tube voltage 120 kV, tube current 250 mA, matrix 512 × 512, tube ball rotation time ≤ 1 s, delayed scanning for 5 s, dynamic scanning for 50 s, a layer thickness of 10 mm, 14 layers, and finally 420 whole brain perfusion images were obtained. The NeroPerfusion software package (Aquarius i Ntuition Edition Ver 4.4.6, Tyrus Imaging Company, USA) provided by the Perfusion Workstation was used to evaluate the data from perfusion imaging. The basilar artery and superior sagittal sinus were selected as the input artery and output vein, respectively. The dose length product (DLP) of a patient was 803.04 mGy·cm (13).

### Imaging Analysis

#### Evaluation of ICA Stenosis

Evaluation of ICA stenosis by DSA: According to the North American Symptomatic Carotid Endarterectomy Trial (NASCET) (14), the ICA stenosis rate was measured and divided into four grades: Grades I (70–79% stenosis rate), II (80–89% stenosis rate), III (90–99% stenosis rate), and IV (100% stenosis rate).

#### Measurement of CTP Parameters of Regions of Interest

Regions of interest (ROIs): ROI measurements were based on the two types of internal watershed infarction (IWI) described by classical neuropathological studies, an infarction involving the corona radiata and the other involving the centrum semiovale (15). Because stenosis of the middle cerebral artery (MCA) was more commonly associated with the corona radiata, the centrum semiovale, which is the first layer after the disappearance of bilateral ventricles, was selected for measurement on CTP imaging. The ROIs excluded the following: blood vessels, calcifications, and necrotic tissues.

**Figure 2:**
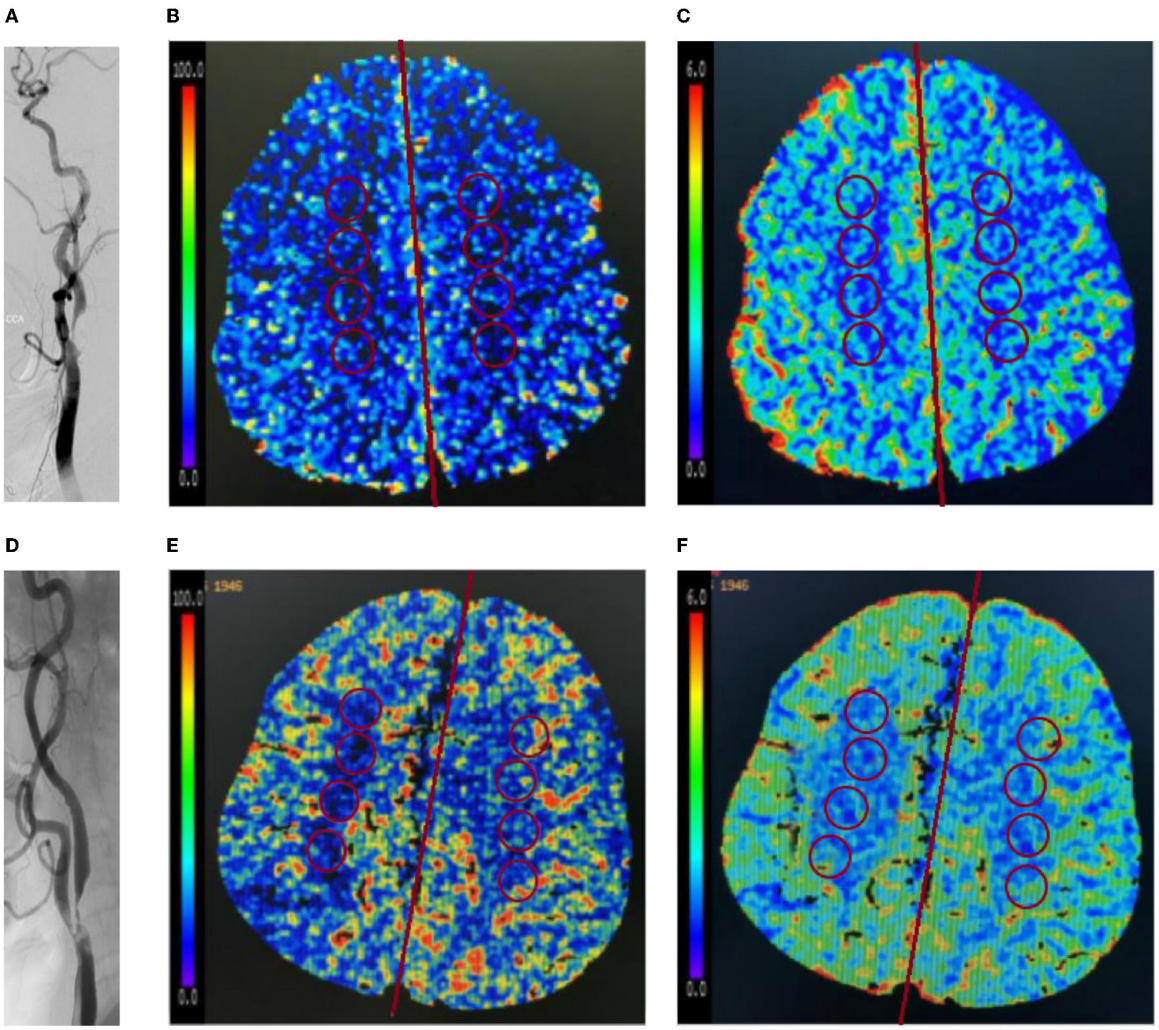
Representative images of patients without or with impaired perfusion. **(A–C)** A 65-year-old female patient was hospitalized with mild headache and was diagnosed as severe stenosis of the right internal carotid artery (ICA) by digital subtraction angiography (DSA) **(A)**. The patient had no impaired perfusion. In the right internal watershed, cerebral blood flow (CBF) **(B)** decreased slightly, cerebral blood volume (CBV) **(C)** increased slightly, and CBF/CBV > 7.55/min. **(D–F)** A 73-year-old male patient was hospitalized with poor left limb activity, and was diagnosed as severe stenosis of the right ICA by DSA **(D)**. The patient had impaired perfusion. In the right internal watershed, CBF **(E)** decreased, and CBV **(F)** decreased slightly, and CBF/CBV < 7.55/min.

CTP parameters included CBF and CBV. Multiple ROIs (each ROI, 2 cm^2^) located within the centrum semiovale on the affected side were selected synchronously on the CBF and CBV parameter map, the midline of the brain was considered as the center line, and the symmetrical ROIs on the healthy side were automatically generated using the CTP software (Figure 2) (16–18). The mean value was calculated as the final CTP parameter value of the corresponding ROI.

In contrast to CBF and CBV taken separately, CBF/CBV would be a more reliable indicator to evaluate the state of cerebral perfusion. The decreased CBF/CBV (<7.55/min) in hemispheres with ICA steno-occlusive disease was categorized as impaired perfusion, which is consistent with the previous research results (7, 8). Based on the “penumbral hypothesis” (8, 19), the patients were divided into two groups: those with impaired perfusion group (CBF/CBV < 7.55/min) and those without impaired perfusion group (CBF/CBV > 7.55/min). The images were independently analyzed by two senior neuroradiologists who were blinded to all the clinical and procedural information of the patients (NZ and MQ). Figure 2 shows ROI measurements of images of a typical patient without impaired perfusion and a typical patient with impaired perfusion.

#### Definitions of Circle of Willis Morphology

The computed tomography perfusion source imaging (CTP-SI) evaluation of the circle of Willis was based on the classification criteria reported by Hartkamp et al. (20). The classification included four types of the circle of Willis: type I, complete anterior and posterior circulation; type II, incomplete posterior circulation and complete anterior circulation; type III, incomplete anterior circulation and complete posterior circulation; and type IV, incomplete anterior and posterior circulation. If the diameters of the first segment of the anterior cerebral artery (ACA-A1) and the first segment of the posterior cerebral artery (PCA-P1) were <1 mm, indicating its inability to act as collateral circulation, we regarded it as absent. The segments of the anterior communicating artery (ACoA) and posterior communicating artery (PCoA) that were not visible on the CTP-SI were considered as missing (21).

A fetal-type posterior (FTP) cerebral artery is defined as a PCoA originating from the ICA and extending directly to the posterior communicating segment of the ipsilateral posterior cerebral artery (PCA), and the diameter of the PCoA is larger than the P1 segment of the ipsilateral PCA originating from the basilar artery (22).

**Figure 3:**
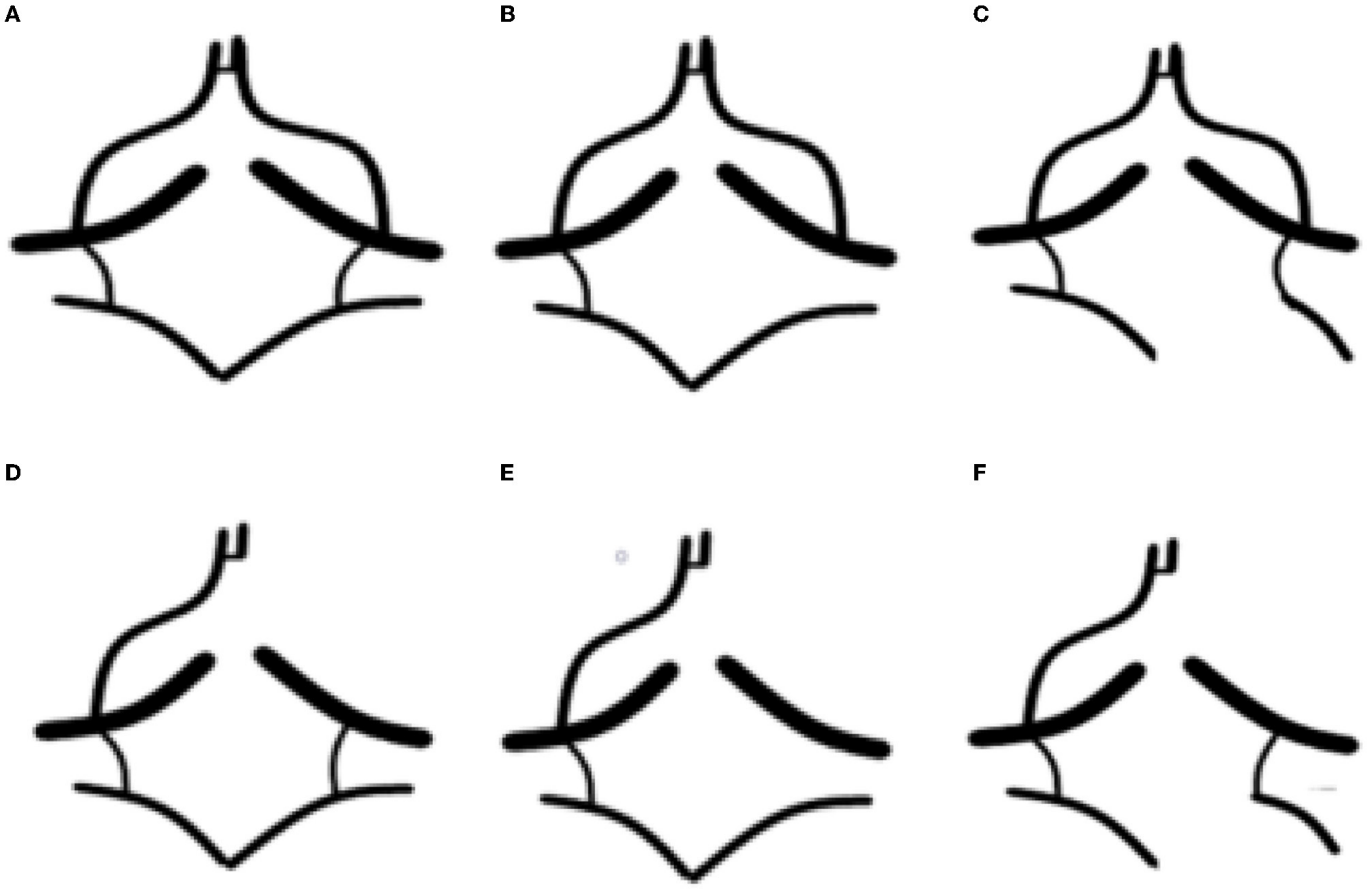
The morphology variants of the circle of Willis in a patient with severe stenosis of left ICA. **(A)** Complete circle of Willis (type I); **(B)** no ipsilateral posterior communicating artery (PCoA) (type II); **(C)** ipsilateral fetal-type posterior cerebral artery (FTP) (type II); **(D)** no ipsilateral in the first segment of the anterior cerebral artery (ACA-A1) (type III); **(E)** no ipsilateral PCoA and incomplete anterior circulation (type IV); and **(F)** ipsilateral fetal-type posterior (FTP) and incomplete anterior circulation (type IV).

According to a previous study (23), the variants of the circle of Willis were further divided into the following six subtypes: complete circle of Willis, no ipsilateral PCoA, ipsilateral FTP, no ipsilateral ACA-A1, no ipsilateral PCoA and incomplete anterior circulation, ipsilateral FTP, and incomplete anterior circulation. Other deformities were not considered. Figure 3 shows the morphology variants of the circle of Willis.

### Statistical Analysis

Data were analyzed by using the Statistical Package for Social Sciences for Windows, Version 20 (IBM Corporation, Armonk, NY, USA). Normally distributed data were described as means and standard deviation (SD). Categorical variables were described as frequency or percentage. Independent sample *t*-test was used for comparisons of measurements that were consistent with normal distribution. The Pearson chi-squared test and Fisher's exact test were used to analyze categorical variables. Univariate and multivariate logistic regression analyses were applied for CTP parameters in healthy and affected sides. Univariate and multivariate logistic regression analyses were applied for CTP parameters and clinical data in the symptomatic group and the asymptomatic group. Multiple logistic stepwise regression analysis was performed to identify independent risk factors associated with impaired perfusion. Univariate analysis of variance (ANOVA) was used to compare the differences of the CBF/CBV value among different vascular variants of the circle of Willis, and then the least significant difference (LSD) method was used for multiple pairwise comparisons. Interobserver agreement was assessed using a single measure statistic (intraclass correlation efficient, ICC). ICC > 0.75 was considered as excellent (24). Statistical significance was defined when *p* < 0.05.

## Results

In this study, there were 187 patients (men, 167) with unilateral ICA steno-occlusive disease (mean age, 65 ± 8 years). Among them, 88 were in the group without impaired perfusion (34 were symptomatic), and 99 were in the group with impaired perfusion (75 were symptomatic).

Of 109 symptomatic patients, the mean CBF of affected and healthy sides was 10.30 ± 3.07 and 14.55 ± 4.07 ml/100 g/min; the mean CBV of affected and healthy sides was 1.54 ± 0.49 and 1.54 ± 0.49 ml/100 g; and the mean CBF/CBV of affected and healthy sides was 6.85 ± 1.44/min and 9.74 ± 2.07/min, respectively.

Of 78 asymptomatic patients, the mean CBF of affected and healthy sides was 13.00 ± 3.93 and 15.23 ± 4.39 ml/100 g/min; the mean CBV of affected and healthy sides was 1.43 ± 0.34 and 1.50 ± 0.41 ml/100 g; and the mean CBF/CBV of affected and healthy sides was 9.22 ± 2.98/min and 10.43 ± 2.56/min, respectively.

The degree of agreement between readers was excellent for CBF (ICC = 0.957) and CBV (ICC = 0.960).

### Univariate and Multivariate Analyses of Factors Associated With Impaired Perfusion

Univariate analysis identified the differences between the two patient groups (without**/**with impaired perfusion), with respect to admission BP (SBP, DBP, and MAP), types of the circle of Willis, and clinical features (*p* < 0.05). No differences in age, sex, hypertension, hyperlipidemia, HHcy, diabetes, CHD, AF, stroke, current smoking status, alcohol use, ICA stenosis rate, and LVEF were found between the two groups (*p* > 0.05) (Table 1).

**Table 1:** Univariate analysis of factors associated with impaired perfusion.

	**Without impaired perfusion** **(*n* = 88)**	**With impaired perfusion** **(*n* = 99)**	***t*/χ^2^**	***P*-value**
Age	66 ± 8	64 ± 7	1.787	0.076
Male gender	81 (92.0%)	86 (86.9%)	1.307	0.253
Hypertension	59 (67.0%)	67 (67.7%)	0.008	0.927
Hyperlipidemia	40 (45.5%)	44 (44.4%)	0.019	0.890
HHcy	19 (21.6%)	32 (32.3%)	2.705	0.100
Diabetes	34 (38.6%)	34 (34.3%)	0.371	0.542
CHD	13 (14.8%)	20 (20.2%)	0.945	0.331
AF	3 (3.4%)	5 (5.1%)		0.725
Stroke history	21 (23.9%)	17 (17.2%)	1.289	0.256
Current smoking	58 (65.9%)	74 (74.7%)	1.753	0.186
Alcohol use	36 (40.9%)	53 (53.5%)	2.978	0.084
**Admission BP**				
SBP	144 ±16	135 ± 18	3.525	0.001
DBP	84 ± 9	79 ± 9	3.583	<0.001
MAP	104 ± 10	98 ± 11	4.024	<0.001
LVEF	0.63 ± 0.03	0.64 ± 0.03	−1.948	0.053
**ICA stenosis rate**			3.761	0.288
Grade I	24 (27.3%)	18 (18.2%)		
Grade II	24 (27.3%)	23 (23.2%)		
Grade III	26 (29.5%)	35 (35.4%)		
Grade IV	14 (15.9%)	23 (23.2 %)		
**Type of circle of Willis**			13.024	0.005
Type I	30 (34.1%)	16 (16.2%)		
Type II	31 (35.2%)	35 (35.4%)		
Type III	15 (17.0%)	16 (16.2%)		
Type IV	12 (13.6%)	32 (32.3%)		
**Clinical features**			26.405	<0.001
Asymptomatic	54 (61.4%)	24 (24.2%)		
Symptomatic	34 (38.6%)	75 (75.8%)		

*HHcy, hyperhomocysteinemia; CHD, coronary heart disease; AF, atrial fibrillation; BP, blood pressure; SBP, systolic blood pressure; DBP, diastolic blood pressure; MAP, mean arterial pressure; LVEF, left ventricular ejection fraction*.

Multiple logistic stepwise regression analysis showed that admission MAP [odds ratio (OR) = 0.946, 95% confidential interval (CI) = 0.917–0.974, *p* < 0.001] and the type of circle of Willis [type IV (type I vs. type IV: OR = 4.987, 95% CI = 1.955–12.723, *p* = 0.001)] were independently associated with impaired perfusion of the internal watershed (Table 2).

**Table 2:** Logistic regression analysis of factors associated with impaired perfusion.

	***P*-value**	**OR value**	**95% CI**	
MAP	<0.001	0.946	0.917	0.974
**Type of circle of Willis**				
Type I vs. type II	0.055	2.190	0.982	4.884
Type I vs. type III	0.160	1.998	0.761	5.243
Type I vs. type IV	0.001	4.987	1.955	12.723

*OR, odds ratio; 95% CI, 95% confidential interval; MAP, mean arterial pressure*.

### Comparison of the CTP Parameters Between Healthy and Affected Sides

From the univariate and multiple logistic regression analyses, CBF and CBF/CBV in the affected side of symptomatic patients were significantly lower than those in the healthy side of symptomatic patients (OR = 0.807, 95% CI = 0.723–0.902, *p* < 0.001; OR = 0.408, 95% CI = 0.309–0.539, *p* < 0.001) (Table 3).

**Table 3:** Comparison of computed tomography perfusion (CTP) parameters between healthy and affected sides.

	**Univariable analysis**		**Multivariable analysis**	
	**OR (95% CI)**	***P-*value**	**OR (95% CI)**	***P-*value**
**Symptomatic patients**				
CBF, mL/100 g/min	0.699 (0.631, 0.773)	<0.001	0.807 (0.723, 0.902)	<0.001
CBV, mL/100 g	1.055 (0.620, 1.795)	0.843		
CBF/CBV, per min	0.369 (0.286, 0.478)	<0.001	0.408 (0.309, 0.539)	<0.001
**Asymptomatic patients**				
CBF, mL/100 g/min	0.919 (0.839, 1.008)	0.072		
CBV, mL/100 g	0.641 (0.258, 1.591)	0.338		
CBF/CBV, per min	0.919 (0.809, 1.044)	0.192		

### Univariate and Multivariate Analyses of Factors Associated With Symptomatic Patients

The CBF/CBV of the affected side is lower in the symptomatic group than in the asymptomatic group (OR = 0.513, 95% CI = 0.393–0.670, *p* < 0.001) (Table 4). Univariate and multiple logistic regression suggested that decreased CBF/CBV was independently associated with symptomatic patients (OR = 0.528, 95% CI = 0.416–0.669, *p* < 0.001) (Table 5).

**Table 4:** Comparison of CTP parameters between asymptomatic and symptomatic patients.

	**Univariable analysis**		**Multivariable analysis**	
	**OR (95% CI)**	***P-*value**	**OR (95% CI)**	***P-*value**
**The healthy side**				
CBF, mL/100 g/min	1.023 (0.944, 1.108)	0.585		
CBV, mL/100 g	1.293 (0.658, 2.543)	0.456		
CBF/CBV, per min	0.963 (0.839, 1.106)	0.595		
**The affected side**				
CBF, mL/100 g/min	0.792 (0.719, 0.873)	<0.001	0.929 (0.821, 1.050)	0.237
CBV, mL/100 g	2.027 (0.953, 4.311)	0.066		
CBF/CBV, per min	0.484 (0.377, 0.622)	<0.001	0.513 (0.393, 0.670)	<0.001

**Table 5:** Univariate and multivariate analysis of factors associated with symptomatic patients.

	**Univariable analysis**		**Multivariable analysis**	
	**OR (95% CI)**	***P-*value**	**OR (95% CI)**	***P-*value**
Age	1.008 (0.970, 1.047)	0.683		
Male gender	0.687 (0.271, 1.740)	0.428		
Hypertension	0.781 (0.418, 1.461)	0.440		
Hyperlipidemia	1.435 (0.796, 2.586)	0.229		
HHcy	1.627 (0.830, 3.189)	0.157		
Diabetes	0.856 (0.469, 1.565)	0.614		
CHD	1.124 (0.521, 2.422)	0.766		
AF	1.202 (0.279, 5.185)	0.805		
Stroke history	0.856 (0.418, 1.756)	0.672		
Current smoking	1.332 (0.713, 2.489)	0.369		
Alcohol use	1.206 (0.673, 2.161)	0.529		
**Admission BP**				
SBP	0.997 (0.981, 1.014)	0.736		
DBP	0.970 (0.940, 1.001)	0.055		
MAP	0.982 (0.956, 1.009)	0.192		
LVEF	0.002 (0.000, 30.489)	0.210		
**ICA stenosis rate**	1.194 (0.903, 1.579)	0.214		
Grade I				
Grade II				
Grade III				
Grade IV				
**Type of circle of Willis**	1.389 (1.055, 1.828)	0.019	1.176 (0.856, 1.615)	0.318
Type I				
Type II				
Type III				
Type IV				
CBF/CBV	0.517 (0.409, 0.655)	<0.001	0.528 (0.416, 0.669)	<0.001

*HHcy, hyperhomocysteinemia; CHD, coronary heart disease; AF, atrial fibrillation; BP, blood pressure; SBP, systolic blood pressure; DBP, diastolic blood pressure; MAP, mean arterial pressure; LVEF, left ventricular ejection fraction*.

**Figure 4:**
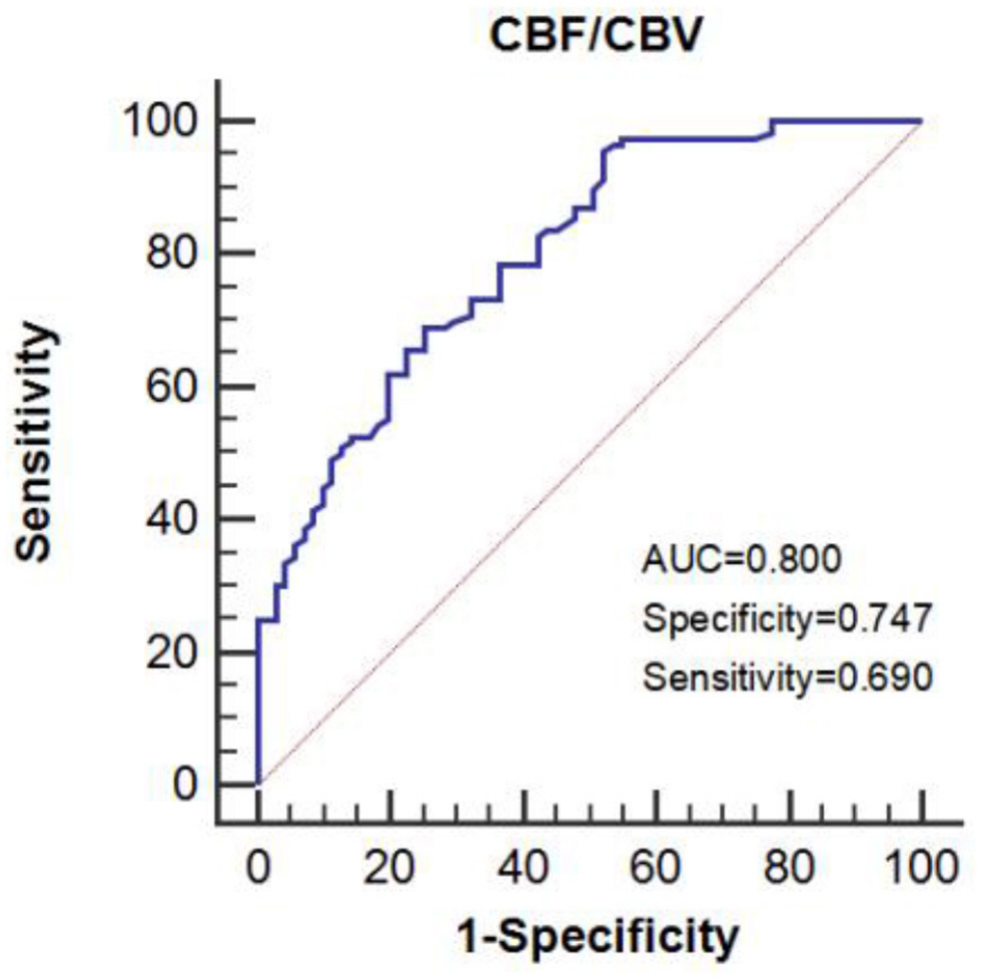
The area under the receiver operating characteristic (ROC) curve predicts impaired perfusion in patients with symptomatic ICA steno-occlusive disease.

The CBF/CBV value of 7.55/min was the best cut-off value for predicting symptomatic patients as shown by the receiver operating characteristic (ROC; area under the curve (AUC): 0.800, sensitivity: 0.690, and specificity: 0.747) (Figure 4).

### CBF/CBV Value and Different Variants of the Circle of Willis

**Figure 5:**
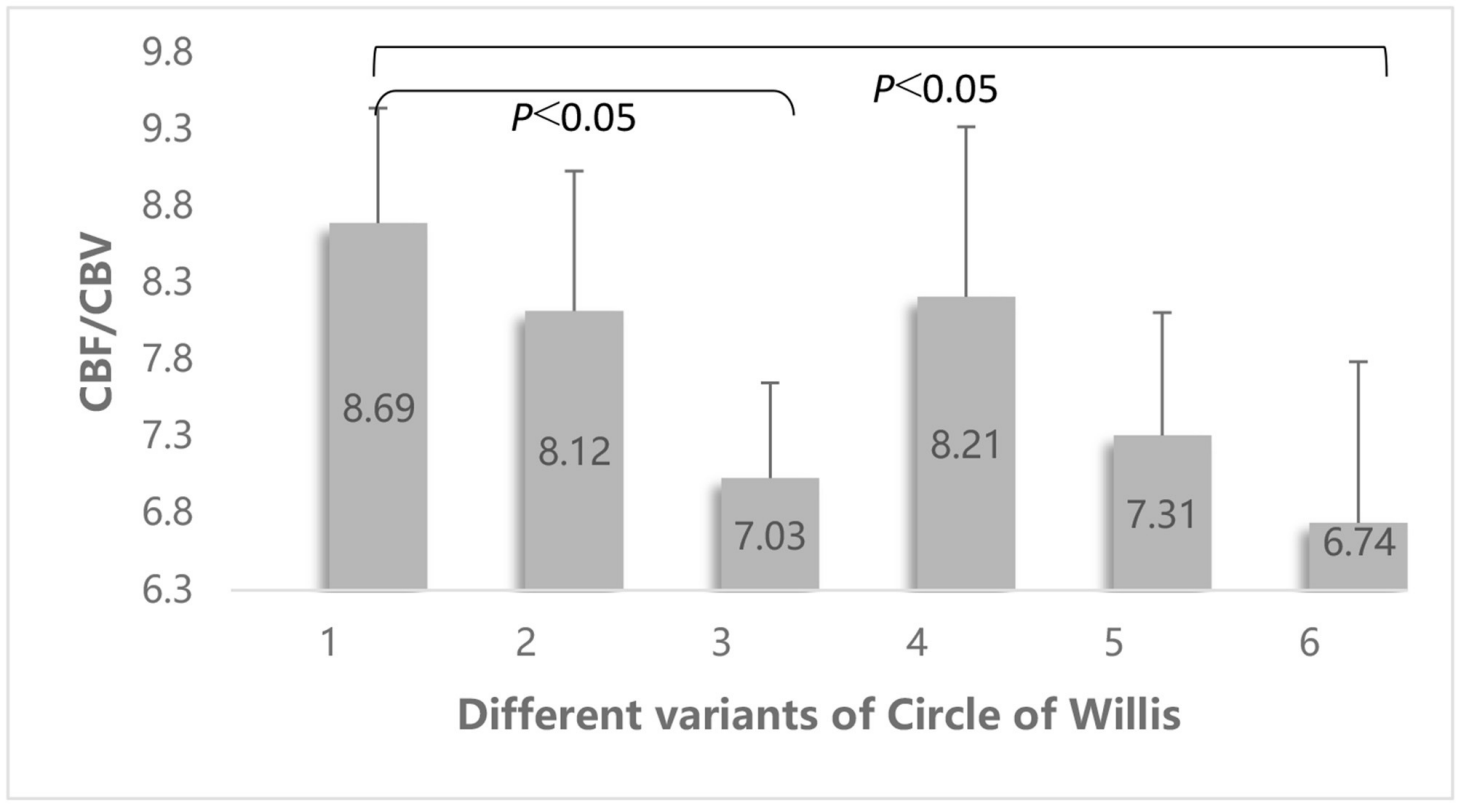
Comparison of the differences of the CBF/CBV value among different vascular variants of the circle of Willis. Different vascular variants of the circle of Willis: 1 = no variation; 2 = no ipsilateral PCoA; 3 = ipsilateral FTP; 4 = no ipsilateral ACA-A1; 5 = no ipsilateral PCoA and incomplete anterior circulation; 6 = ipsilateral FTP and incomplete anterior circulation.

Univariate ANOVA showed that there were significant differences in the CBF/CBV of patients with different vascular variants of the circle of Willis (*F* = 3.131, *p* = 0.010). Pairwise comparisons indicated that the CBF/CBV in patients with an ipsilateral FTP and patients with an ipsilateral FTP and incomplete anterior circulation was significantly lower than that in patients with a complete circle of Willis (7.03 ± 1.69 vs. 8.69 ± 2.53, *p* = 0.004 and 6.74 ± 2.37 vs. 8.69 ± 2.53, *p* = 0.002) (Figure 5).

## Discussion

The evaluation of the status of cerebral perfusion in patients with a stenotic or occluded ICA before treatment is essential. A few studies have shown that patients with impaired perfusion in the hemisphere ipsilateral to a stenotic ICA may have an increased risk of ischemic stroke than those with normal perfusion (25, 26). Our study found that in symptomatic patients CBF and CBF/CBV in the affected hemisphere was lower than that in the healthy hemisphere. Compared with asymptomatic patients, the CBF/CBV value of symptomatic patients with unilateral ICA steno-occlusive disease decreased correspondingly. Our study also found that lower MAP levels on admission and the type of circle of Willis (type IV) were independent risk factors of impaired perfusion when the unilateral ICA was severely stenotic or occluded. Moreover, we also assessed the FTP variants that were associated with decreased CBF/CBV.

In this study, impaired perfusion in the internal watershed area was associated with decreased MAP levels. This finding is consistent with that of Yamauchi's finding that effective BP management could facilitate reperfusion and play a protective role in ischemic brain tissue (7). As an index of local cerebral perfusion pressure, CBF/CBV is directly related to the systemic MAP. Within a certain MAP range, the relationships between CBF/CBV and MAP appear to be linear (8). A decrease in BP may directly lead to a further decrease in the perfusion of the cerebrum that was originally hypoperfused, thus eventually lead to cerebral ischemia or infarction, especially in the internal watershed area that was already sensitive to changes in BP. A few studies have shown that BP management is a vital component of the treatment of hypoperfusion cerebral infarction (27–29). However, in these studies, the specific range of BP that is considered moderate was not clearly defined. Using the Cox regression analysis, Wohlfahrt (30) found that patients with an admission MAP < 100 mm Hg may be at an increased risk of hemodynamic stroke. Maddula (31) reported that the maintenance of a SBP ranging 130–150 mm Hg was effective in avoiding cerebral hypoperfusion.

From the findings of our study, we think that impaired perfusion was not associated with the ICA stenosis rate, but impaired perfusion was associated with the type of circle of Willis (type IV) and FTP. Thus, we hypothesized that when the unilateral ICA was severely stenosed or occluded, the restricted ipsilateral carotid blood flow might be compensated by the circle of Willis and an adaptive expansion of cerebral vessels. The carotid stenosis rate may not fully reflect the cerebral perfusion situation or an ischemic event (32). As we know, the internal watershed area is located deep in the white matter, which is in the terminal zone of the ICA, the compensation provided by the leptomeningeal collateral circulation is insufficient, which is mainly compensated by the primary collateral circulation. Whenever the ICA is stenosed or occluded, a circle of Willis with an abnormal morphology increases the risk of cerebral ischemia or infarction (33, 34). Theoretically, a complete circle of Willis can reperfuse the ischemic region before irreversible damage occurs. The compensatory effect on the internal watershed areas remains uncertain because of insufficient research on this area. Our study found that an excellent primary collateral circulation was protective of the internal watershed area, and that decreased CBF/CBV in the internal watershed area was strongly associated with type IV circle of Willis.

Medullary arterioles in the watershed areas showed insufficient self-regulation, and there was no compensatory dilation of pial vessels. If a patient had an incomplete circle of Willis, the redistribution of blood flow would be affected and result in a markedly negative effect (35).

However, variants of the circle of Willis are diverse and the compensatory capacity of different variations also varies. Therefore, we also evaluated the effect of single-vessel variation on CBF/CBV. In this study, we suggest that the presence of an FTP, with or without an incomplete anterior circulation, was a risk factor for impaired perfusion in the internal watershed area. On the single side of the FTP, the ICA supplies blood to both MCA and PCA, which affects the onset of a secondary collateral flow between the ICA and vertebrobasilar arterial system. This leads to a bilaterally imbalanced CBF, and the perfusion pressure of the affected side is relatively lower than that of the opposite side.

Patients with unilateral ICA steno-occlusive disease, combined with an ipsilateral FTP, have been reported to exhibit a marked reduction in the ipsilateral cerebral perfusion pressure. In addition, they are more likely to show decompensation and infarction involving watershed areas (36). Bisschops (37) concluded that an ACoA deficiency increased the incidence of IWI in patients with a stenotic ICA. This event might be related to the poor collateral compensation between the cortical branch of the MCA and the lenticular artery or between the MCA and superficial cortical branches of the ACA. However, unlike in our study, the FTP was not analyzed by Bisschops.

Previous studies have reported that ICA with severe stenosis or occlusion combined with elevated blood viscosity and an elevated risk of clotting may lead to a cerebral watershed infarction (CWSI) (15). However, in our study, there were no significant differences in the degree of hyperlipidemia and HHcy between the two groups. Probably, these factors promoted the occurrence of atherosclerosis by increasing the changes of blood viscosity but did not directly affect cerebral perfusion in our study.

Our results demonstrate that quantitative evaluation of the cerebral perfusion status and risk factors of impaired perfusion in patients with symptomatic unilateral ICA steno-occlusive disease is clinically relevant. If a subgroup of high-risk patients could be correctly identified by clinical doctors, the risk of irreversible cerebral infarction could be reduced. In addition, relevant studies have found that, when brain tissues have weak tolerance to ischemia and hypoxia, it is more likely to have more severe cerebral ischemia or infarction under the influence of ischemic intervention factors (38).

This study had some limitations. The study's sample size was relatively small, and the enrollment bias could not be avoided. A large sample in multiple centers is needed in future studies. Moreover, this study did not consider the effect of mild cerebral vascular stenosis on cerebral perfusion. Finally, BP was measured once at admission and included in the analysis, making it difficult to exclude the fluctuation of BP. A 24-h ambulatory BP should be considered for future studies.

In conclusion, admission MAP and the type of circle of Willis were found to be independent risk factors associated with the internal watershed impaired perfusion in patients with unilateral ICA steno-occlusive disease. Variants in the FTP were also associated with decreased CBF/CBV.

## Conclusion

MAP and the type of circle of Willis at admission may be independent risk factors associated with the impaired perfusion in patients with ICA steno-occlusive disease.

## Data Availability Statement

The raw data supporting the conclusions of this article will be made available by the authors, without undue reservation.

## Ethics Statement

The studies involving human participants were reviewed and approved by the Institutional Review Board of General Hospital of Northern Theater Command. The patients/participants provided their written informed consent to participate in this study. Written informed consent was obtained from the individual(s) for the publication of any potentially identifiable images or data included in this article.

## Author Contributions

XQ, JD, and ZX conceived the project idea and wrote the manuscript. XW and YD provided critical suggestions for the design of experiments. YP, NZ, MQ, and JL collected the imaging and clinical data. ZX and BY supervised the project. All authors contributed to this article and approved the submitted version.

## Funding

This study was supported by Grant No. 202054044 from the Project of Natural Science Foundation of Shenyang and by Grant No. 201602768 from the Project of Natural Science Foundation of Liaoning province.

## Conflict of Interest

The authors declare that the research was conducted in the absence of any commercial or financial relationships that could be construed as a potential conflict of interest.

## Publisher's Note

All claims expressed in this article are solely those of the authors and do not necessarily represent those of their affiliated organizations, or those of the publisher, the editors and the reviewers. Any product that may be evaluated in this article, or claim that may be made by its manufacturer, is not guaranteed or endorsed by the publisher.
